# Surgical Approaches in Total Hip Arthroplasty: Does the Choice Between Direct Anterior and Direct Lateral Affect Blood Loss?

**DOI:** 10.7759/cureus.103582

**Published:** 2026-02-14

**Authors:** Yuval Fuchs, Tal Shachar, Alex Tavdi, Refael Behrbalk, Guy Shabtai, Shanny Gur, David Segal, Nissim Ohana, Yaron Brin

**Affiliations:** 1 Orthopaedics, Meir Medical Center, Kfar Saba, ISR; 2 Orthopaedic Surgery, Meir Medical Center, Kfar Saba, ISR

**Keywords:** anemia, blood loss, direct anterior, direct lateral approach, hemoglobin, total hip arthroplasty

## Abstract

Background

Total hip arthroplasty (THA) is the treatment of choice for end-stage hip osteoarthritis and displaced femoral neck fractures in the active elderly. While the direct anterior (DA) and direct lateral (DL) approaches are commonly used in THA, the impact of these approaches on blood loss remains a subject of debate. This study aims to compare the extent of blood loss associated with the DA and DL approaches in THA.

Methods

We conducted a retrospective review of 278 patients who underwent THA using either the DA or DL approach at a single medical center between January 2014 and October 2020. Hemoglobin (Hb) levels were measured preoperatively and on postoperative day 1 (POD1) to assess blood loss. We also collected data on demographic variables, comorbidities, operative time, and other relevant clinical factors. Statistical analyses were performed to evaluate differences between the two surgical approaches.

Results

The mean postoperative reduction in Hb levels was -2.49 ± 1.11 g/dL in the DA group, and -2.25 ± 1.14 g/dL in the DL group, with no statistically significant difference observed (p = 0.069). Although the DL approach was associated with longer operative times (p = 0.003), both approaches resulted in comparable blood loss. When stratified by surgical indication, a statistically significant difference in Hb loss was found only in trauma patients, with greater loss in the DA group (p = 0.048). Despite these findings, the clinical significance of these differences remains limited.

Conclusions

Our study suggests that the choice between the DA and DL approaches does not significantly impact blood loss in THA. The findings indicate that surgical expertise and patient-specific factors should guide the selection of the surgical approach, rather than concerns over blood loss alone. Further research with larger, more diverse cohorts is needed to validate these findings and explore other potential benefits or drawbacks of each approach.

## Introduction

Total hip arthroplasty (THA) is a highly effective surgical intervention for patients suffering from severe osteoarthritis that impairs daily functioning, as well as those with displaced subcapital fractures. As it is cost-effective and has a high patient satisfaction rate [1], THA has become a commonly performed procedure [2] involving the replacement of both the femoral and acetabular components. Although there is widespread agreement on the indications for THA, consensus on the optimal surgical approach remains elusive. The three main approaches for THA include the postero-lateral (PL) approach, the direct lateral (DL) approach, and the direct anterior (DA) approach. While the DA becomes associated with good patient functional outcome [3,4] and less pain [5], it requires a slow learning curve [6]. Furthermore, evidence regarding hemoglobin (Hb) changes postoperatively remains insufficient.

A decreased Hb level following hip surgery in trauma patients has been linked to reduced walking distances at discharge and an increased risk of postoperative delirium [7].

A study investigating the impact of anemia on post-operative functional mobility has found that anemia is significantly associated with decreased functional mobility in the early post-operative phase [8]. Additionally, preoperative anemia was prevalent in approximately 24% of the patients undergoing elective THA/total knee arthroplasty and 51% of the patients undergoing surgical hip fracture repair [9].

Patient blood management interventions aimed at reducing the need for allogeneic blood transfusions and improving patient outcomes warrant increased medical attention.

The aim of this study was to evaluate the influence of surgical approach (DA vs. DL) on perioperative blood loss, as assessed by postoperative Hb decline (delta Hb) and transfusion rates, in patients undergoing THA.

## Materials and methods

This is a retrospective, single-center study that was conducted from January 1, 2014, to October 1, 2020. Inclusion criteria for the study were patients who underwent THA using either the DA or DL approach. Patients who underwent THA for indications other than primary osteoarthritis or subcapital fractures were excluded from the analysis.

Patients

In this study, only active patients who underwent THA for either hip osteoarthritis or a femoral neck fracture were included based on the National Institute for Health and Care Excellence (NICE) guidelines [10] included in the study. Patients were divided into two cohorts according to the surgical approach: DL or DA. In addition to stratification by surgical approach (DA vs. DL), patients were further stratified according to surgical indication: primary osteoarthritis (non-trauma) vs. subcapital fracture (trauma). This stratification was performed to evaluate potential differences in perioperative Hb change and operative characteristics between trauma and non-trauma cohorts.

Surgical approach

Direct Anterior Approach

Patients were positioned supine on a standard operating table capable of flexing at the hip. A modified Hueter approach was employed to access the hip joint. The surgery began with a transverse skin incision on the anterior thigh, starting 5 cm below the anterior superior iliac spine and extending up to 10 cm laterally along the anterior thigh. The DA approach involved exposing the tensor fascia lata (TFL) muscle and dividing its perimysium. The interval between the sartorius and TFL muscles was utilized for access. The lateral head, or reflected portion, of the rectus femoris muscle was retracted medially. An anterior capsulectomy was performed to expose the femoral neck. The femoral neck was then osteotomized, and the femoral head and neck were removed using a corkscrew. The acetabulum and femoral canal were subsequently prepared in a routine manner. Exposure of the femoral canal involved selective soft tissue releases on the posterior aspect of the femoral neck while preserving the abductor mechanism and the short external rotators.

Direct Lateral Approach

Patients were positioned in a lateral decubitus position on a regular operating table. The DL was performed by placing the incision over the greater trochanter and dividing the underlying fascia lata. The abductor mechanism was divided into its middle third, retracting the anterior half anteriorly. A capsulotomy was performed, and the femoral neck fracture was exposed. A femoral neck osteotomy was performed, and the femoral head and neck were removed. The acetabulum and femoral canal were prepared in the conventional manner.

Implants

All patients underwent THA using the Corail cementless stem and Pinnacle cementless cup (DePuy Synthes, Warsaw, IN) with the same surgical team.

Patient's data

Hb levels were measured for all patients scheduled for THA, both preoperatively and on postoperative day 1 (POD1) onwards. To assess the differences in Hb levels, we calculated the gap between preoperative and postoperative values, referred to as "Delta Hb." The potential need for postoperative blood transfusion was determined only after reviewing the Hb levels on POD1, and all transfusions administered within the study group were recorded. Additionally, data on variables such as comorbidities, duration of surgery, type of anesthesia, and patient demographics were collected for each participant. All patients received tranexamic acid according to our institutional protocol, administered as 500 mg orally three times during the first 24 hours postoperatively (total dose 1,500 mg).

Surgeons

All surgeries in this study were performed by the same surgical team, ensuring consistency in technique and minimizing variations in outcomes. All procedures were performed by senior arthroplasty surgeons, each with over seven years of specialized experience in hip reconstruction and a cumulative experience of several hundred THA procedures.

Statistical analysis

Descriptive statistics were used to summarize baseline and perioperative characteristics. Continuous variables are presented as mean ± standard deviation (SD), and categorical variables as frequencies and percentages. For univariate comparisons, the chi-square test was used for categorical variables and a two-sided Student’s t-test for continuous variables. Stratified analyses according to surgical indication (primary osteoarthritis vs. trauma) were pre-specified and performed to evaluate potential differences within these clinically distinct subgroups.

No formal multivariable regression model was performed. Given the retrospective design, an a priori power calculation was not conducted, and the sample size reflects all eligible consecutive patients during the study period. No formal adjustment for multiple comparisons was applied; therefore, subgroup findings, particularly borderline p-values, should be interpreted with caution. A p-value < 0.05 was considered statistically significant. Statistical analyses were performed using IBM SPSS Statistics (version 28, IBM Corp., Armonk, NY).

Stratification parameters

For subgroup analyses, patients were stratified according to predefined clinical and perioperative parameters. Stratifications included surgical approach (DA vs. direct DL), indication for surgery (primary osteoarthritis vs. subcapital fracture), and transfusion requirement (yes/no). Additional stratifications were performed based on demographic and clinical variables, including age, sex, comorbidities, type of anesthesia, and duration of surgery. Hb dynamics were further evaluated using delta Hb (preoperative minus postoperative Hb level), allowing comparison of perioperative blood loss patterns between groups. These stratifications were selected a priori to assess their potential association with postoperative Hb decline and transfusion rates.

Ethical considerations

Patient data were collected using our electronic medical records. The study was approved by our Institutional Review Board and waived written informed consent requirements. Generative AI or large language models were not used in the writing process or text revision.

## Results

We reviewed a total of 388 medical records, of which 278 patients met the inclusion criteria. The mean age of the patients was 68.4 ± 9.4 years. There were no significant differences in age or gender distribution between the two surgical approach groups. Hip osteoarthritis was the primary indication for surgery in 147 patients, while intracapsular femoral neck fracture was the leading cause in the remaining 131 patients (Table 1).

**Table 1:** Characteristics and univariable analysis of patients OA, osteoarthritis; Tx, treatment

Variable	Direct anterior n = 155 (55.8%)	Direct lateral n = 123 (44.2%)	Total n = 278 (100%)	p-value
Age (mean ± SD)	69.36 ± 8.7	67.2 ± 10.05	68.41 ± 9.37	0.056
Sex (male n, %)	65 (41.9%)	49 (39.8%)	114 (41%)	0.409
Indication				0.362
OA	80 (51.6%)	67 (54.5%)	147 (52.9%)	-
Trauma	75 (48.4%)	56 (45.5%)	131 (47.1%)	-
Side (right n, %)	77 (49.7%)	72 (58.5%)	149 (53.6%)	0.088
Hg level change (g/dL mean ± SD)	-2.49 ± 1.11	-2.25 ± 1.14	-2.38 ± 1.13	0.069
Hg level - preoperative (g/dL mean ± SD)	13.24 ± 1.49	13.14 ± 1.38	13.2 ± 1.44	0.56
Hg level - first postoperative (g/dL mean ± SD)	10.74 ± 1.4	10.89 ± 1.19	10.81 ± 1.31	0.35
Hg level change (g/dL mean ± SD)				
OA (n, mean ± SD)	n = 80: -2.43 ± 1.04	n = 67: -2.32 ± 1.12	2.38 ± 1.18	0.577
Trauma (n, mean ± SD)	n = 75: -2.57 ± 1.17	n = 56: -2.16 ± 1.17	2.39 ± 1.19	0.048
Anesthesia				<0.001
Complete (n, %)	66 (42.6%)	18 (14.6%)	84 (30.2%)	-
Spinal (n, %)	88 (56.8%)	101 (82.1%)	189 (68%)	-
Both (n, %)	1 (0.6%)	4 (3.3%)	5 (1.8%)	-
Operation time - from "first cut" (hours, mean ± SD)	1:44:0:50 ± 22	1:59:29 ± 0:55	1:51 ± 0:40	0.003
Anticoagulation Tx (n, %)	12 (7.7%)	5 (44.1%)	17 (6.1%)	0.154
Operation time - from "first cut" (hours, mean ± SD)				
OA (n, mean ± SD )	n = 80: 1:45:33 ± 0.20	n = 67: 1:54:09 ± 0:32	n = 147: 1:49:27 ± 0:27	0.055
Trauma (n, mean ± SD)	n = 75: 1:44:04 ± 0:24	n = 56: 2:06 ± 1:14	n = 131: 1:53:12 ± 0:52	0.018
Cement (n, %)	4 (2.6%)	3 (2.4%)	7 (2.5%)	0.626
Hypertension (n, %)	69 (44.5%)	61 (49.6%)	130 (46.8%)	0.235
Dyslipidemia (n, %)	49 (31.6%)	56 (45.5%)	105 (37.8%)	0.017
Diabetes mellitus (n, %)	34 (21.9%)	35 (28.5%)	69 (24.8%)	0.211
Lung disease (n, %)	15 (9.7%)	13 (10.6%)	28 (10.1%)	0.806
Coagulopathy (n, %)	6 (3.9%)	3 (2.4%)	9 (3.2%)	0.503
Vasculopathy (n, %)	12 (7.7%)	8 (6.5%)	20 (7.2%)	0.692
Cardiac pathologies (n, %)	26 (16.8%)	17 (13.8%)	43 (15.5%)	0.499
Blood transfusion (n, %)	34 (21.9%)	23 (18.7%)	57 (20.5%)	0.507

**Figure 1:**
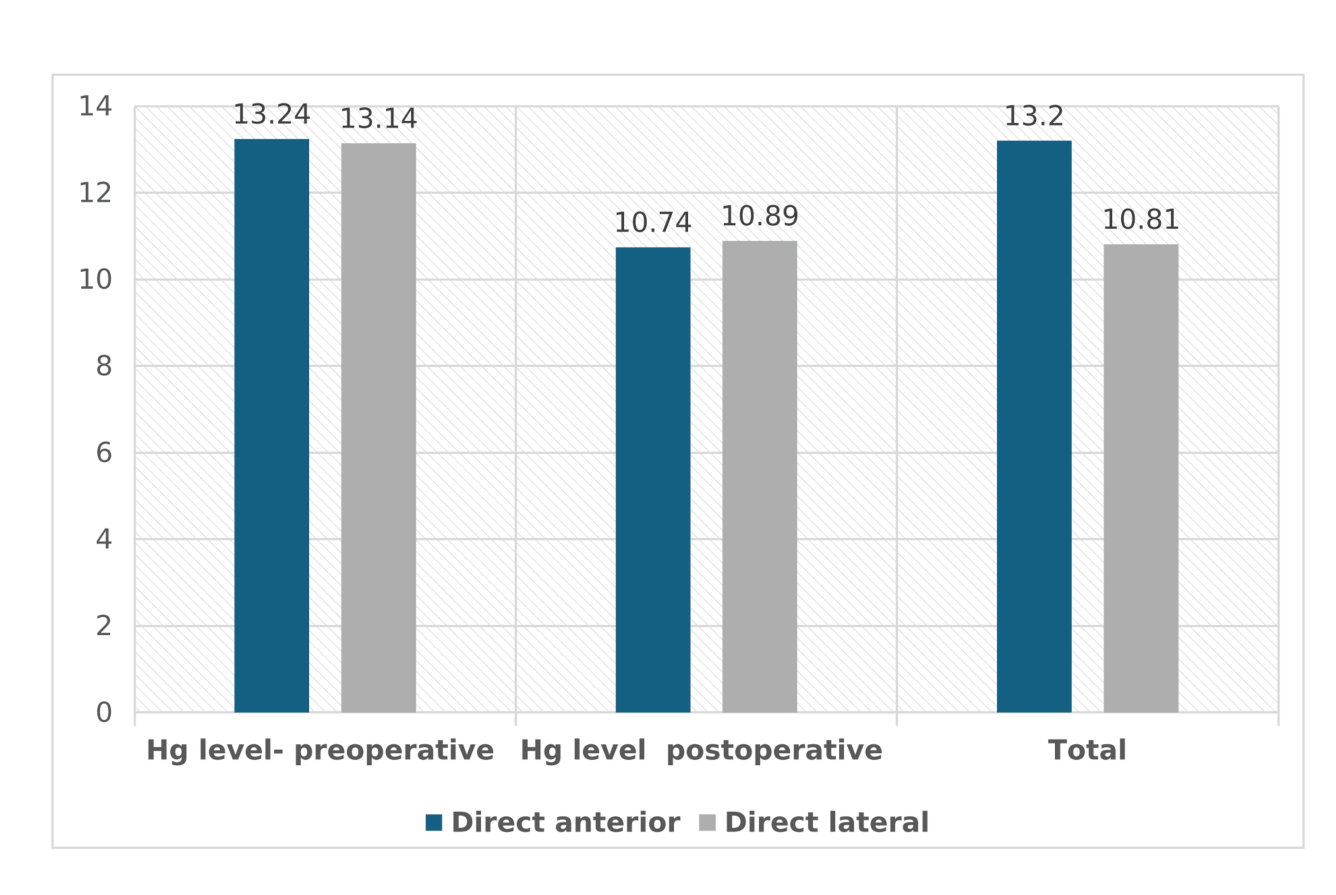
Schematic presentation of hemoglobin loss

Preoperative Hb levels were similar between the DA and DL approach groups (13.24 ± 1.49 g/dL vs. 13.14 ± 1.38 g/dL, respectively; p = 0.56). Postoperative Hb levels also showed similar results (10.74 ± 1.4 g/dL vs. 10.89 ± 1.19 g/dL, respectively; p = 0.35) (Figure 1). The mean postoperative reduction in Hb level was -2.38 ± 1.13 g/dL for the entire cohort, with no significant difference between the DA and DL approaches (p = 0.069). However, the DL approach showed a slightly smaller mean Hb level change (-2.25 ± 1.14 g/dL) compared to the DA approach (-2.49 ± 1.11 g/dL). When stratified by indication, a statistically significant difference was observed in the mean change of Hb levels in trauma patients. Those undergoing the DA approach experienced a greater mean decrease in Hb levels (-2.57 ± 1.17 g/dL) compared to those undergoing the DL approach (-2.16 ± 1.17 g/dL) (p = 0.048).

The mean age was 69.36 years in the DA group and 67.31 years in the DL group. The mean operation time was significantly longer for patients who underwent the DL approach (one hour, 59 minutes, and 29 seconds ± 55 seconds; p = 0.003). Regarding comorbidities, hypertension was the most common, affecting 130 patients (46.8%), with no significant difference in its distribution between the two approaches (p = 0.235). Dyslipidemia was more prevalent in the DL approach group (56 patients, 45.5%) compared to the DA group (49 patients, 31.6%) (p = 0.017). There were no significant differences between the two approaches in the prevalence of diabetes mellitus, lung disease, coagulopathy (chronic kidney disease, hemophilia, or use of anticoagulation therapy), vasculopathy, cardiac pathologies, or the need for blood transfusion.

## Discussion

Various surgical techniques for THA have been developed over the years as the search for the ideal surgical approach continues. When considering recovery following THA, surgeons must weigh factors such as intraoperative blood loss, recovery time, and overall patient outcomes when deciding on the surgical approach.

The DA approach, known for its muscle-sparing technique, often facilitates quicker recovery and reduces postoperative pain, potentially leading to shorter hospital stays [11]. Conversely, the more traditional DL approach provides excellent visibility and control during surgery, which can be particularly beneficial in complex cases [12]. While many studies have discussed the functional outcomes of these surgical approaches, there is limited information available regarding the extent of Hb loss associated with each approach. This study aims to explore the differences between the DA and DL approaches in the context of THA for subcapital femoral neck fractures and osteoarthritis, with an emphasis on reduced blood loss.

Duration of surgery is a known risk factor for blood loss and infection, with a shorter operative time being associated with a lower risk of postoperative infection. According to Wang et al. [13], for each 20-minute increase in operative time during joint replacement procedures, there is a 25% increase in the risk of postoperative infection. Our results show that patients in the DL group had significantly longer operative times (one hour, 59 minutes, and 29 seconds ± 55 seconds) compared to the DA group (one hour, 44 minutes, and 33 seconds ± 22 seconds; p = 0.003). One possible explanation is that the DL approach requires more time for the closure of its soft tissue layers. Other studies have shown conflicting results regarding operative time. While a recent randomized prospective study [14] found no difference in operative time between DA and DL approaches, others [15,16] reported a difference inoperative time between the two approaches.

When stratified by surgical indication, our study shows the DA approach is associated with a significantly shorter operative time compared to the DL approach in the trauma cohort (p = 0.018). Furthermore, Hb loss was found to be different between DA and DL only in the trauma cohort (p = 0.048). Although these findings may suggest that the surgical approach, particularly in the context of trauma, can be influenced by the surgical approach, the clinical relevance of this change may be questionable and should be treated with caution.

The mean age of patients in our study was slightly higher in the DA group (69.36 ± 8.7 years) compared to the DL group (67.2 ± 10.05 years). Though this difference was not statistically significant (p = 0.056), age is an important factor to consider when selecting a surgical approach. As older patients often present with multiple comorbidities that can both complicate surgery and impact recovery rates and time [17-19], a shorter procedure might therefore be beneficial.

The Hb loss observed during the DA approach may be attributed to the challenging exposure and release of the posterior capsule [20]. However, it is important to note that the use of the intermuscular plane in the DA causes less muscle damage as indicated by lower levels of inflammatory markers and creatine kinase when compared to the posterior approach [21]. Although muscle damage, as measured by serum markers and MRI, is less prevalent in DA patients than in DL patients, this difference does not translate into functional outcomes within the first three postoperative months [22].

Maezawa et al. [23]. evaluated muscle strength after THA by calculating the 10 days postoperative/preoperative Hb ratio and measuring the strength leg rise (SLR). Their findings suggest that patients with an Hb ratio greater than 85% have higher SLR strength, indicating that postoperative hip function may be influenced by Hb levels.

Postoperative anemia is a common phenomenon following orthopedic surgeries [9] and is known to negatively impact the recovery phase by reducing patient mobility [8], hip strength [23], and increasing the risk of infection. Previous reports have shown a relationship between anemia and postoperative recovery, with anemia impeding functional mobility and affecting daily activities in patients with hip osteoarthritis or those who have undergone THA. The study also found that a higher postoperative Hb level was associated with better postoperative rehabilitation and improvement in daily activities [19].

While allogenic blood transfusion is a common method used to treat anemia [24], it can also lead to potential risks and complications such as transfusion-related acute lung injury (TRALI) and hemolytic transfusion reactions [25].

Given that post-operative anemia increases the risks of morbidity and mortality, we chose to compare the change in Hb levels between the two cohorts (DA and DL) to better understand the likelihood of additional bleeding. This comparison aims to determine whether surgeons should consider blood loss when planning THA using either surgical approach.

Our findings revealed no statistically significant difference in Hb drop between the DA and DL approaches. Specifically, the mean Hb decrease was -2.49 ± 1.11 mmHg in the DA group and -2.25 ± 1.14 mmHg in the DL group (p = 0.069). This suggests that, with modern surgical techniques and perioperative management, the blood loss associated with both approaches is comparable.

Our results align with those of both Parvizi et al. [14] and Restrepo et al. [26], whose prospective randomized study reported no difference in blood loss between the DA and DL approach. Our findings further support the notion that when contemporary blood conservation strategies are employed, both the DA and DL approaches yield comparable outcomes in terms of blood loss.

While both Barrett et al. [20] and Zhao et al. [27] found increased blood loss in the DA in the short postoperative period, both studies compare DA to the PL approach rather than to the DL, as our study does. Another study by Alecci et al. [15] compared DA to the standard lateral approach (Bauer’s) and found a statistically significant difference in Hb change in favor of the DA approach. However, this comparison was with the standard lateral approach (Bauer’s), not the DL (Hardinge) method used in our study.

In osteoarthritis management, our findings underscore the substantial healthcare and economic benefits; aligning surgical technique with surgeon expertise minimizes blood loss and supports better outcomes. Although Hb changes in trauma cases were statistically significant (p = 0.048), the clinical impact was limited. Surgeons should use the approach they are most proficient in.

Several limitations should be considered when interpreting the findings of this study. First, the retrospective design introduces inherent risks of selection bias and information bias and limits the ability to establish causal relationships between surgical approach and perioperative outcomes. Although consecutive patients were included, unmeasured confounders may still have influenced the results. Second, this was a single-center study conducted within one public hospital, which may limit the external validity and generalizability of the findings. Institutional protocols, perioperative management strategies (including transfusion thresholds and blood conservation protocols), and surgeon-specific technical preferences may differ across centers. While the use of a standardized surgical team and implant system enhances internal consistency, it may reduce applicability to institutions with different practice patterns. Third, although baseline characteristics and comorbidities were compared between groups using univariate analyses and stratification by surgical indication (trauma vs. non-trauma), a formal multivariate regression model adjusting simultaneously for comorbidities and perioperative variables was not performed. Incorporating multivariable analysis could have further clarified the independent effect of surgical approach while controlling for potential confounders such as age, comorbidity burden, anesthesia type, and operative time. Therefore, residual confounding cannot be completely excluded. Finally, the sample size, particularly within subgroup analyses, may limit statistical power and increase the risk of type II error, potentially obscuring smaller but clinically meaningful differences between groups.

## Conclusions

The study's findings suggest that although the DL approach was associated with longer operative times, the similar blood loss observed between the DA and DL approaches indicates that the impact of operative time on blood loss may be less critical than previously thought. This underscores the potential influence of other factors, such as surgical technique and perioperative management, in determining blood loss. However, further research is needed to explore other potential benefits or drawbacks of each approach, particularly in different patient populations or settings. Studies with larger sample sizes and diverse patient groups are necessary to validate and expand upon these findings.
